# The effect of endometrial injury on pregnancy rate in frozen-thawed embryo transfer: A randomized control trial

**Published:** 2016-07

**Authors:** Abbas Aflatoonian, Ramesh Baradaran Bagheri, Robabe Hosseinisadat

**Affiliations:** 1 *Research and Clinical Center for Infertility, Shahid Sadoughi University of Medical Sciences, Yazd, Iran.*; 2 *Department of Obstetrics and Gynecology, School of Medicine, Kerman University of Medical Sciences, Kerman, Iran. *

**Keywords:** *Endometrial injury*, *Frozen-thawed embryo transfer*, *Pipelle catheter*, *Implantation rate*, *Pregnancy rate*

## Abstract

**Background:**

Implantation failure is one of the most important factors limiting success in IVF treatment. The majority of trials have demonstrated favorable effect of endometrial injury on implantation success rate especially in women with recurrent implantation failure, while some studies failed to detect any benefit.

**Objective:**

The purpose of our trial was to explore whether endometrial injury in luteal phase prior to frozen-thawed embryo transfer cycles would improve pregnancy outcomes?

**Materials and Methods:**

We conducted a prospective controlled trial of 93 consecutive subjects at a research and clinical center for infertility. All women were undergone frozen-thawed embryo transfer (FTE) cycles. Women in the experimental group underwent endometrial biopsy with a Pipelle catheter in luteal phase proceeding FET cycle. Primary outcomes were implantation and clinical pregnancy rates and secondary outcomes were chemical, ongoing and multiple pregnancy and miscarriage rates.

**Results:**

45 subjects who underwent endometrial injury (EI) were compared with 48 control group which did not include any uterine manipulation. There were no significant differences in baseline and cycle characteristics between two groups. The difference in implantation rate was trend to statistically significance, 11.8% in EI group vs. 20.5% in control group (p=0.091). The chemical, clinical and ongoing pregnancy rates were lower in EI group compared with control group but not statistically significant. The multiple pregnancy rate and miscarriage rate also were lower in EI group compared with control group.

**Conclusion:**

Based on results of this study, local injury to endometrium in luteal phase prior to FET cycle had a negative impact on implantation and clinical pregnancy rates.

## Introduction

Implantation failure is one of the most important factors limiting success in IVF treatment (1). Embryo implantation is a critical process of embryonic attachment to endometrium and subsequent invasion into uterine wall (2). Uterus is receptive during mid-secretory phase (days 19-23) of menstrual cycle, which is known as window of implantation (2). Implantation of embryo is a multiple process including several cytokines and growth factors, along with a dialogue between embryo and uterine endometrium (3). Numerous factors have been contributed increasing embryo implantation success (4). Majority of trials have demonstrated favorable effect of endometrial injury on implantation success rate, especially in women with recurrent implantation failure (RIF), while some studies failed to detect any benefit (5-13). 

Kalma *et al* suggested that “local injury to endometrium causes significant changes in pattern of expression of genes related to implantation” (14). Gnainsky *et al* reported that “endometrial injury induces an inflammatory reaction which favors implantation” (15). Dendritic cells, natural killer cells and macrophages are employed to local injury and increased amounts of cytokines, chemokines and growth factors are secreted, thus resulting in successful implantation (15, 16). 

To our knowledge, there has not been enough research due to the effectiveness of endometrial injury prior to frozen-thawed embryo transfer (FET) cycle. The purpose of our trial was to explore whether endometrial injury in luteal phase prior to FET cycle would improve pregnancy outcomes?

## Materials and methods

**Study design and participants**

This randomized clinical trial conducted at Research and Clinical Center for Infertility, Yazd, Iran, between March 2015 to January 2016. Ethical confirmation was received from Ethic Committee of Research and Clinical Center for Infertility and written informed consent was obtained from all participants. For study population a computer-generated randomization table was created. 

The inclusion criteria include: women indicated for FET treatment, had one or more frozen embryo(s) and had a normal uterine cavity (confirmed by vaginal ultrasonography). The exclusion criteria were women <40 yrs, history of endocrine disorders (hypothyroidism, diabetes mellitus), intrauterine abnormality (uterine polyp, sub-mucosal fibroma, intrauterine adhesion) and severe endometriosis diagnosed by laparoscopy or endometrioma in ultrasound scanning.

**Figure 1:**
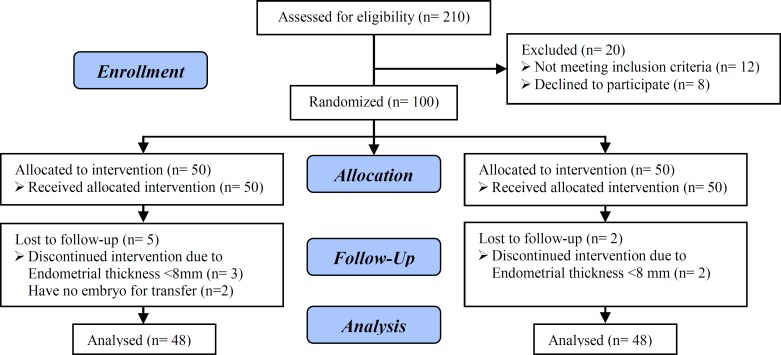
Consort 2010 flow diagram of the study.

This study included initially 120 eligible participants. 20 patients excluded because of not meeting inclusion criteria (n=12) and declining to participate in the study (n=8). We allocated the remaining 100 participants in two groups: endometrial injury (EI) group (n=50) and non-endometrial injury (nEI) group (n=50). Five patients in EI group were excluded because of endometrial thickness <8 mm (n=3) and having no embryos for transfer (n=2). Two patients in nEI group were excluded because of endometrial thickness <8 mm. Finally 45 women in EI group and 48 women in nEI group were analyzed (Figure 1).

In the EI group, women underwent endometrial injury between day 21 and 23 of menstrual cycle proceeding FET cycle. EI was performed in standard fashion using Pipelle catheter (Endobiops, Prince Medical France). Catheter was introduced through the cervix up to uterine fundus. The piston was drawn back to create a negative pressure. Sheath was rotated and moved back and forth 2-3 times before it was withdrawn. In the subsequent cycle, all of women underwent our standard endometrial preparation protocol for FET cycles with estradiol valerate 6 mg daily from day 2 of the cycle. 

A transvaginal ultrasound was then performed in day 13 of cycle and if endometrial thickness was ≥8 mm with a triple-line appearance, subject was started on vaginal progesterone pessary 800 mg daily (Actavis, UK) and embryo transfer was performed 3 days later with 6-8 cell frozen-thawed embryos with COOK catheter (USA) by an expert infertility fellowship.

**Outcome measures**

The primary outcomes were implantation and clinical pregnancy rates and secondary outcomes were chemical, ongoing, and multiple pregnancy and miscarriage rates. Chemical pregnancy rate was defined as positive hCG test 14 days after embryo transfer. Implantation rate was the sacs number seen on transvaginal ultrasound scan divided by the number of transferred embryos. 

Clinical pregnancy rate was defined by ultrasound detection of gestational sac and fetal heart activity approximately 5 wks after embryo transfer. Ongoing pregnancy rate was defined as presence of fetal heart activity on ultrasound beyond 12 wks. Multiple pregnancy rates were defined as the number of multiple pregnancies divided by total number of clinical pregnancies. Miscarriage rate was defined as miscarriages number before 20 wks divided by the number of women with a positive pregnancy test. 

**Sample size calculation**

A power analysis based on Barash *et al *with 30% difference in clinical pregnancy rate, demonstrated that we would require 49 patients per group to give a test with the significance of 5% and a power of 80% in this prospective randomized design (5).

**Statistical analysis**

SPSS software (Statistical Package for the Social Sciences version 20.0, SPSS Inc., Chicago, IL, USA) was used for all statistical calculations. Student’s t-test was used for comparing quantitative variables and ^2^ test used to compare categorical data. p<0.05 was considered statistically significant.

## Results

In total, 93 women who underwent FET treatment were analyzed. Women were divided into two groups: EI (n=45) and nEI group which did not include any uterine manipulation in preceding luteal phase (n=48). Baseline characteristics between two groups were compared (Table I). There were no significant differences in baseline characteristics analyzed including age, type of infertility, duration and causes of infertility and number of previous embryo transfer(s) (Table II). There were no significant differences between two groups including treatment duration, endometrial thickness at progesterone initiation day, number and quality of frozen-thawed embryos transferred. 

Pregnancy outcomes of patients in both groups are shown in table III. Implantation rate was lower in EI (11.8%) compared with nEI group (20.5%), observed difference was trend to statistically significance (p=0.091). Although chemical (26.7% vs. 39.6%), clinical (22.2% vs. 33.3%) and ongoing (22.2% vs. 31.2%) pregnancy rates were lower in EI compared with nEI group, the observed differences were short of reaching statistically significance. Multiple pregnancy (10% vs. 25%) and miscarriage rates (16.7% vs. 21.1%) were lower in EI compared with nEI group with no statistically difference.

**Table I:** Baseline characteristics of patients in both groups

		**EI group (n=45)**	**non-EI group (n=48)**	**p-value**
Age (years)*		32.35 ± 5.61	31.4 ± 4.43	0.251^$^
Duration of infertility (years) *		6.42 ± 3.62	6.33 ± 3.62	0.907 ^$^
Type of infertility **				
	Primary	33 (73.3%)	39 (81.2%)	0.459^#^
	Secondary	12 (26.7%)	9 (18.8%)	
Causes of infertility**				
	Male factor	23 (51.1%)	26 (54.2%)	0.842^#^
	PCO	8 (17.8%)	11 (22.9%)	
	POF	5 (11.1%)	3 (6.2%)	
	Tubal factor	2 (4.4%)	4 (8.3%)	
	Endometriosis	1 (2.2%)	1 (2.1%)	
	Unexplained	2 (4.4%)	1 (2.1%)	
	Mixed	4 (8.9%)	2 (4.2%)	
Number of previous transfer(s) **				
	0	2 (4.4%)	8 (16.7%)	0.163^#^
	1-2	35 (77.8%)	33 (68.8%)	
	3	8 (17.8%)	7 (14.6%)	

* Data are presented as mean±S.D.

** Data presented as n (%).

$ Student t-test

# Chi-square test

**Table II:** Cycle characteristics of patients in both groups

		**EI group (n=45)**	**non-EI group (n=48)**	**p-value**
Treatment duration (days)*		17.48 ± 2.58	17.12 ± 2.94	0.529^$^
Endometrial thickness at progesterone initiation day (mm) *		9.13 ± 1.42	8.60 ± 1.37	0.072 ^$^
Number of transferred embryos *		2.11 ± 0.64	2.16 ± 0.63	0.676 ^$^
Quality of transferred embryos n (%)				
	A	5 (11.1%)	10 (20.8%)	0.194^#^
	B	35 (77.8%)	29 (60.4%)	
	C	5 (11.1%)	9 (18.8%)	

* Data are presented as mean±S.D.

$ Student t-test

# Chi-square test

**Table III:** Pregnancy outcomes of patients in both groups

	**EI group (n=45)**	**non-EI group (n=48)**	**p-value**
Implantation rate*	11.8% ± 20.6%	20.5% ± 27.3%	0.091
Chemical pregnancy rate **	12 (26.7%)	19 (39.6%)	0.187
Clinical pregnancy rate **	10 (22.2%)	16 (33.3%)	0.233
Ongoing pregnancy rate **	10 (22.2%)	15 (31.2%)	0.326
Multiple pregnancy rate **	1 (10%)	4 (25%)	0.429
Miscarriage rate **	2 (16.7%)	4 (21.1%)	0.763

* Data presented as mean±S.D.

** Data are presented as n (%).

## Discussion

In the current study, endometrial injury performed in luteal phase preceding a FET cycle, had a negative impact on implantation and pregnancy outcomes. Previous studies have reported an improvement in clinical pregnancy and/or live birth rates after endometrial injury (1-4). The reported significant benefits in patients with RIF have made it tempting intervention to be offered to all patients prior to their IVF treatments. However, most of studies have been underpowered and there has been very limited data exploring the role of endometrial injury in FET treatment.

The role of endometrial injury in IVF was controversial. Barash *et al* first demonstrated that EI during the cycle preceding IVF doubled the implantation rates , clinical pregnancy, and live birth rates in women with RIF (5). Several studies confirmed the positive effect of EI on embryo implantation and clinical pregnancies at different time and with different frequencies, however, conflicting results were reported (1, 6, 7). Yeung *et al* demonstrated that EI performed in luteal phase of preceding cycle does not improve the ongoing pregnancy rate in unselected subfertile women undergoing IVF (8, 9). 

Therefore, population, timing, technique and frequencies of endometrial injury were variable and led to different outcomes. The mechanism underlying EI action, remains unclear. Another study demonstrated that the implantation success was secondary to the development of an inflammatory reaction induced by trauma (10). It has been supposed that the injury to endometrium induces secretion of cytokines and growth factors that will stay in basal layer of endometrium for a few cycles and enhance decidualization and facilitate implantation (11-15). It has also been demonstrated that endometrial injury up-regulates the gene expression related to endometrial receptivity which optimizes endometrial development (16-18). 

To our knowledge, no study has demonstrated the effectiveness of endometrial injury prior to transferring frozen-thawed embryos. The results of this study suggest that endometrial response to injury during a FET cycle is different, or does not confer the same benefit, as it does during IVF-ET cycle. An explanation for this diversity might be sought in various hypotheses about why endometrial injury is helpful for implantation which mentioned above. An alternate explanation was offered by Zhou *et al* called “backwards development theory”. They speculated that controlled ovarian hyperstimulation (COH) negatively affects embryo implantation through histological progression and functional changes such as pinopode maturation advancement and steroid receptor down-regulation. 

The trauma to endometrium stimulates a wound repair process which creates a lag and serves to better sync the uterus with implanting embryo (19). If the “backwards development theory” explains why patients who have recently undergone COH can benefit from EI, then our results would be expected in FET cycles. It is possible that the frequency and endometrial injures timing , as well as the degree of injury, may have an impact on implantation and pregnancy outcomes. There is no consensus on optimal frequency and timing of procedure(s) required for endometrial injury to induce its maximal effect. Methodological and recruitment differences complicate the results comparison in FET cycles to those previously published for IVF-ET. Original publication by Barash *et al* included 4 biopsies, while other studies have been limited to 1 or 2 (2, 19). 

A detrimental effect has been demonstrated when the endometrial injury was performed in transfer cycle on the oocyte retrieval day (8). In the current study, we performed a single endometrial biopsy in mid-luteal phase prior to FET cycle. This is presumed ‘window of implantation’ with the highest abundance of cytokines and growth factors in endometrium, where the endometrial injury effect, if any, may be maximized (20).

Although the recent systematic reviews and meta-analyses have concluded a beneficial effect of endometrial injury in patients with RIF, they have included non-randomized studies and only a limited number of available randomized trials were included (21, 22). When we review the available RCTs assessing the endometrial injury effect on pregnancy outcomes, most of them either did not have priori sample size calculation or well-defined primary outcome, or they were terminated before completion of recruitment (1-3, 23, 24). 

These factors would have ability limited to draw reliable conclusions with adequate power. One of the limitations of current study was the absence of placebo and both our physicians and patients were not blinded to randomization. However, due to intervention nature , the physicians could not be blinded and patients would likely be aware of intervention.

## Conclusion

In summary we concluded that EI in luteal phase prior to FET cycle did not improve implantation nor did it improve clinical pregnancy rates. Indeed we found that EI in luteal phase prior to FET cycle had a negative impact on implantation and pregnancy outcomes. 

Currently, there is lack of good evidence to support routine endometrial injury prior to FET treatment. The lower multiple pregnancy and miscarriage rates in EI group would be a benefit effect of endometrial injury in FET treatment. The large randomized controlled trial of FET cycles might need to define the mechanism by which EI is helpful for IVF-ET cycles and if this can be applied to other treatments for infertility.

## Note

***Registration ID in IRCT: IRCT2015101324512N1***
