# Correction to “The effect of endometrial injury on pregnancy rate in frozen-thawed embryo transfer: A randomized control trial” [Int J Reprod BioMed 2016; 14: 453-458]

**DOI:** 10.18502/ijrm.v20i3.10715

**Published:** 2022-04-21

**Authors:** Abbas Aflatoonian, Ramesh Baradaran Bagheri, Robabe Hosseinisadat

The authors of article entitled ``The effect of endometrial injury on pregnancy rate in frozen-thawed embryo transfer: A randomized control trial" have requested some corrections in their article due to further analysis of their data. The authors reviewed the data and confirmed the critical but inadvertent statistical analysis errors which had occurred during the research. As the authors explained in their letter to the editor, the errors are listed as:

• The recruitment end date has been changed from January 2016 to December 2015.
• The Fisher's exact test has been added in the statistical analysis section.
• A typographical error occurred in the eligible woman which was mistyped as 210 instead of 120. Also, in the first group, data on 45 women were analyzed in the end, but 48 was incorrectly stated.
• Some numbers in the tables have been changed and statistical tests have been revised as follows:

**Table 1:** Baseline characteristics of patients in both groups


		**EI group (n = 45)**	**Non-EI group (n = 48)**	**P-value**
Age (yr)*		32.35 ± 5.61	31.4 ± 4.43	0.366 ${}^{\$}$
Duration of infertility (yr)*		6.42 ± 3.62	6.33 ± 3.62	0.025 ${}^{\$}$
Type of infertility**			
	** Primary**	33 (73.3)	39 (81.2)	0.362 ${}^{*}$
	** Secondary**	12 (26.7)	9 (18.8)	
Causes of infertility**				
	** Male factor**	23 (51.1)	26 (54.2)	0.842 ${}^{\#}$
	** PCO**	8 (17.8)	11 (22.9)	
	** POF**	5 (11.1)	3 (6.2)	
	** Tubal factor**	2 (4.4)	4 (8.3)	
	** Endometriosis**	1 (2.2)	1 (2.1)	
	** Unexplained**	2 (4.4)	1 (2.1)	
	** Mixed**	4 (8.9)	2 (4.2)	
Number of previous transfer(s)**				
	** 0**	2 (4.4)	8 (16.7)	0.163 ${}^{\#}$
	** 1-2**	35 (77.8)	33 (68.8)	
	** 3**	8 (17.8)	7 (14.6)	
*Data are presented as Mean ± S.D. **Data presented as n (%). ${}^{\$}$ Student *t* test, ${}^{\#}$ Chi-square test, *Fisher exact test				

**Table 3:** Pregnancy outcomes of patients in both groups


	**EI group (n = 45)**	**Non-EI group (n = 48)**	**P-value**
Implantation rate*	11.8 ± 20.6	20.5 ± 27.3	0.085
Chemical pregnancy rate**	12 (26.7)	19 (39.6)	0.187
Clinical pregnancy rate**	10 (22.2)	16 (33.3)	0.233
Ongoing pregnancy rate**	10 (22.2)	15 (31.2)	0.326
Multiple pregnancy rate**	1 (10.0)	4 (25.0)	0.61***
Miscarriage rate**	2 (16.7)	4 (21.1)	1.00***
*Data presented as Mean ± S.D. **Data are presented as n (%). ***Fisher exact test			

One of the readers of the article informed the corresponding author that there seemed to be a number of errors in presenting the data, so the authors re-analyzed the data and due to the selection of an inappropriate statistical test, some numbers were incorrect. The corrected article has been provided with corrections to the paper and relevant tables. The authors have confirmed that there are no other errors. The corrected article has been reviewed by our editorial team, and we have confirmed that the overall conclusion has not been changed as stated in the updated article available at: DOI: 10.18502/ijrm.v14i7.768 (updated on March 3, 2022).

